# Seasonal Changes in Thyrotropin and Thyroid Hormones in Women of Reproductive Age

**DOI:** 10.3390/life15030365

**Published:** 2025-02-26

**Authors:** Sevgül Fakı, Abbas Ali Tam, Pervin Demir, Didem Özdemir, Fatma Neslihan Çuhacı Seyrek, Ekin Yiğit Köroğlu, Oya Topaloğlu, Reyhan Ersoy, Bekir Çakır

**Affiliations:** 1 Department of Endocrinology and Metabolism, Ankara Bilkent City Hospital, 6800 Ankara, Turkey; eyigitkoroglu@hotmail.com; 2 Department of Endocrinology and Metabolism, Faculty of Medicine, Ankara Yıldırım Beyazıt University, 6800 Ankara, Turkey; 3 Department of Biostatistics and Medical Informatics, Faculty of Medicine, Ankara Yıldırım Beyazıt University, 6800 Ankara, Turkey

**Keywords:** thyroid hormones, reproductive age, seasonal changes, thyroid stimulating hormone

## Abstract

Background: Thyroid hormones are essential for the proper functioning of the female reproductive system. Seasonal changes in thyroid hormones in women of reproductive age are not fully understood. we aimed to investigate seasonal changes in thyrotrophin and thyroid hormones in women of reproductive age. Methods: Approximately 75,000 TSH and thyroid hormone levels measured in 32,935 women between 2019 and 2023 were evaluated on a monthly and seasonal basis. The analysis of means (ANOM) method was utilized to compare the mean transformed rank of each group or the overall mean transformed rank for TSH, FT3, and FT4 values across months and seasons. Results: The differences from the maximum to minimum median values were 13.3% for TSH, 5.2% for free T3, and 5.9% for free T4. TSH levels were significantly lower in summer and autumn and higher in winter and spring than the overall transformed mean. The rate of individuals with TSH levels below 2.5 mIU/L was higher in summer and autumn than in spring and winter. Conclusions: TSH varies seasonally in women of reproductive age. It is appropriate to consider the season as well as other parameters that affect thyroid functions while evaluating thyroid hormones.

## 1. Introduction

Thyroid hormones are essential for normal reproductive function, and triiodothyronine (T3) and thyroxine (T4) modulate the development and metabolism of the ovaries, uterus, and placenta via interaction with specific receptors in these organs [1]. Thus, subfertility, infertility, menstrual irregularities, anovulation, abortion, and intrauterine developmental delay may be seen due to thyroid dysfunctions [1,2]. These adverse events may also occur to a lesser extent in borderline abnormalities such as subclinical hypothyroidism and hypothyroxinemia [3].

Although thyroid dysfunctions can be seen at any period of life, clinical or subclinical hypothyroidism is seen at a rate of 0.3% in women of reproductive age and 4.3% during pregnancy [1,4,5]. Fetal thyroid hormone secretion begins at around 14–18 weeks of pregnancy. Thyroid hormones are essential for fetal growth and brain development, especially during early pregnancy, when the fetus is entirely dependent on the mother [6]. Thyroid hormone deficiency in the mother in the first trimester can affect gross motor skills, visual processing, visual attention, and even the memory of the offspring [7].

TSH measurement is essential both for monitoring thyroid diseases and for monitoring hormone control in patients with hypo- or hyperthyroidism who are receiving drug therapy. Serum TSH concentrations can be affected by age, gender, iodine intake, and cigarette smoking [8]. In addition, seasonal changes may be observed in TSH levels, and TSH tends to increase in winter [8,9,10]. However, seasonal changes are not taken into account for thyroid hormone reference range values [11]. In this context, ignoring TSH changes in individuals over time may lead to under or overdiagnosing (subclinical) thyroid diseases. Management of subclinical hypothyroidism can be challenging because TSH levels may return to normal [12] without any intervention.

There are very few studies on seasonal changes in TSH in women of reproductive age. Effective management of thyroid disease before pregnancy is critical because the devastating effects of thyroid dysfunctions can be prevented with appropriate treatment. Therefore, using the big data approach, we aimed to investigate the monthly and seasonal changes in TSH and thyroid hormones in women of reproductive age for four consecutive years.

## 2. Materials and Methods

The medical records of female patients aged 18–49 who applied to our outpatient clinic between February 2019 and May 2023 were evaluated retrospectively. Inpatients and pregnant women were excluded from this study. Patients were included in this study regardless of whether they had thyroid disease or were on medication.

Blood samples for TSH, free T3 (FT3), and free T4 (fT4) were taken between 08:00 a.m. and 4:00 p.m. These parameters were determined by an immunoassay analyzer with a chemiluminescence method using acridinium ester technology (Atellica IM 1600 Analyzer, Siemens Healthcare Diagnostics, Erlangen, Germany). The normal ranges for TSH, FT3, and FT4 were 0.55–4.78 mU/L, 2.3–4.2 ng/L, and 0.89–1.78 ng/dL, respectively. Ankara, where this study was conducted, is at an altitude of 891 m, at 39.9727’ latitude and 32.8637’ longitude, and is a city where the temperature drops below 0 °C degrees in winter and can rise to 30–40 °C in summer. The assay used for TSH measurement remained unchanged for a period of four years. Both intra- and interassay variability were assessed. For internal quality control, Siemens conducts intra-assay controls twice daily, and the results are evaluated in accordance with Westgard rules. Interassay variability was analyzed on a monthly basis. The laboratory used is a member of the Bio-Rad organization. The biochemistry laboratory where hormone measurements were performed is maintained at a constant temperature of 20 °C throughout the year, regardless of it being summer or winter, via a central air conditioning system. The heat emitted by the equipment is compensated for by the ventilation system, ensuring that the ambient temperature remains stable.

TSH, FT3, and FT4 levels were compared between months and between seasons. March, April, and May were defined as spring, June, July, and August as summer, September, October, and November as autumn, and December, January, and February as winter.

Subclinical hypothyroidism and subclinical hyperthyroidism were defined as high and low TSH values, respectively, in the presence of normal FT3 and FT4. Overt hypothyroidism was defined as high TSH with a low level of thyroid hormones, and overt hyperthyroidism was defined as low TSH with a high level of thyroid hormones. Our center is a multi-faceted referral center that accepts individuals with many different conditions, ranging from thyroid dysfunctions (hypo-hyperthyroidism), to nodular thyroid disease, to health check-ups. With this big data approach, we conducted our study with a large dataset of patients over the age of 18 (including both patients and healthy individuals) who applied to our center regardless of whether they were using drugs that affect or could potentially affect thyroid functions.

According to the TSH levels, the patients were divided into two groups (TSH ≤ 2.5, TSH > 2.5), and seasonal ratios were compared.

### Statistical Analysis

Quantitative variable distributions were assessed using Shapiro–Wilk’s test, complemented by histogram, Q-Q Plot, and box-plot graphs. Summary statistics for quantitative and qualitative variables included the mean ± standard deviation or median (25th–75th percentiles) and frequency (%). For values below/above the defined detectible limits in TSH, FT3, and FT4, imputation was carried out using the “zCompositions” and “robCompositions” R packages. Imputation for censored values involved non-parametric spline smoothing (Kaplan–Meier spline smoothing) and Tobit regression methods. The Kruskal–Wallis non-parametric variance test was employed to compare concentration values between months or seasons. In the case of significance, the stepwise stepdown comparison method by Campbell and Skillings was applied. Due to the right-skewed distribution, a logarithmic transformation was performed before conducting covariance analysis (ANCOVA) with age adjustments. The analysis of means (ANOM) method was utilized to compare the mean transformed rank of each group or the overall mean transformed rank for TSH, FT3, and FT4 values across months and seasons. This analysis was conducted using the “ANOM” R packages. The transformation of values was achieved through the formula Φ^−1^[0.5 + (ranked value/(2n + 1))]). Significance was determined when the transformed rank mean deviated outside the upper definition limes (UDLs) and lower definition lines (LDLs). LASSO regression was employed using the “caret” and “glmnet” packages in R software (Version 4.4.1) to address multicollinearity and enhance prediction accuracy in a regression model. LASSO introduces penalties on independent variable coefficients, preventing overfitting by penalizing the absolute values of the coefficients. In LASSO regression, *p*-values for coefficients are not directly available, as it does not provide traditional statistical tests for variable significance. Instead, the magnitude of the coefficient indicates the strength of the relationship, with larger magnitudes suggesting a more substantial impact on the dependent variable. The analysis utilized a cosinor model, incorporating trigonometric functions (cosine and sine components) to capture seasonal variations, providing a comprehensive approach to modeling temporal effects. All additional calculations were conducted using SPSS version 21.0 (Armonk, NY, USA: IBM Corp.). The statistical significance threshold for all analyses was set at *p* < 0.05.

The effect size (f) for monthly and seasonal differences, calculated from retrospectively collected data of 32,935 women, ranged from 0.056 (Cohen’s d = 0.111) to 0.159 (Cohen’s d = 0.316). A post hoc power analysis indicated a statistical power of 0.999 across all effect sizes.

Local ethical committee approval was obtained on 11 November 2023 (E1-23-4213), in accordance with the ethical standards of the Declaration of Helsinki. Appropriate processes were followed during the research. Informed consent was obtained from all individual participants included in this study.

## 3. Results

The TSH values were obtained from 32,935 women with a mean age of 36.28 ± 8.57 and median age of 37 (min-max: 18–49 years). The medians for 75,581 TSH, 75,567 FT3, and 75559 FT4 serum samples were 1.70 mU/L (Q1–Q3: 0.68–3.00), 3.23 ng/L (Q1–Q3: 2.93–3.56), and 1.15 ng/dL (Q1–Q3: 1.03–1.30), respectively.

### 3.1. Monthly Variations in the Median TSH, FT3, and FT4 Levels

**Figure 1:**
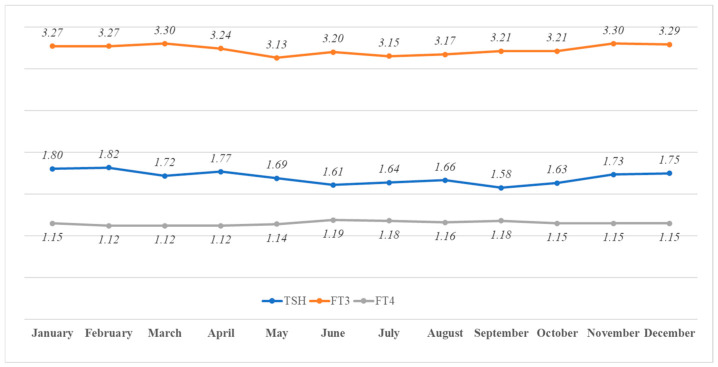
The monthly median TSH (mIU/L), FT3 (ng/L), and FT4 (ng/dL) concentrations in each month.

Serum TSH, FT3, and FT4 were significantly different between months (*p* < 0.001 for each) (Figure 1). After conducting the ANCOVA, with the effects of age controlled for, these differences were still present (*p* < 0.001 for TSH, FT3, and FT4). The differences from the maximum to minimum median values were 13.3% for TSH, 5.2% for FT3, and 5.9% for FT4.

### 3.2. Transformed Rank Mean Comparison in Each Month for TSH, FT3, and FT4 Levels

**Figure 2:**
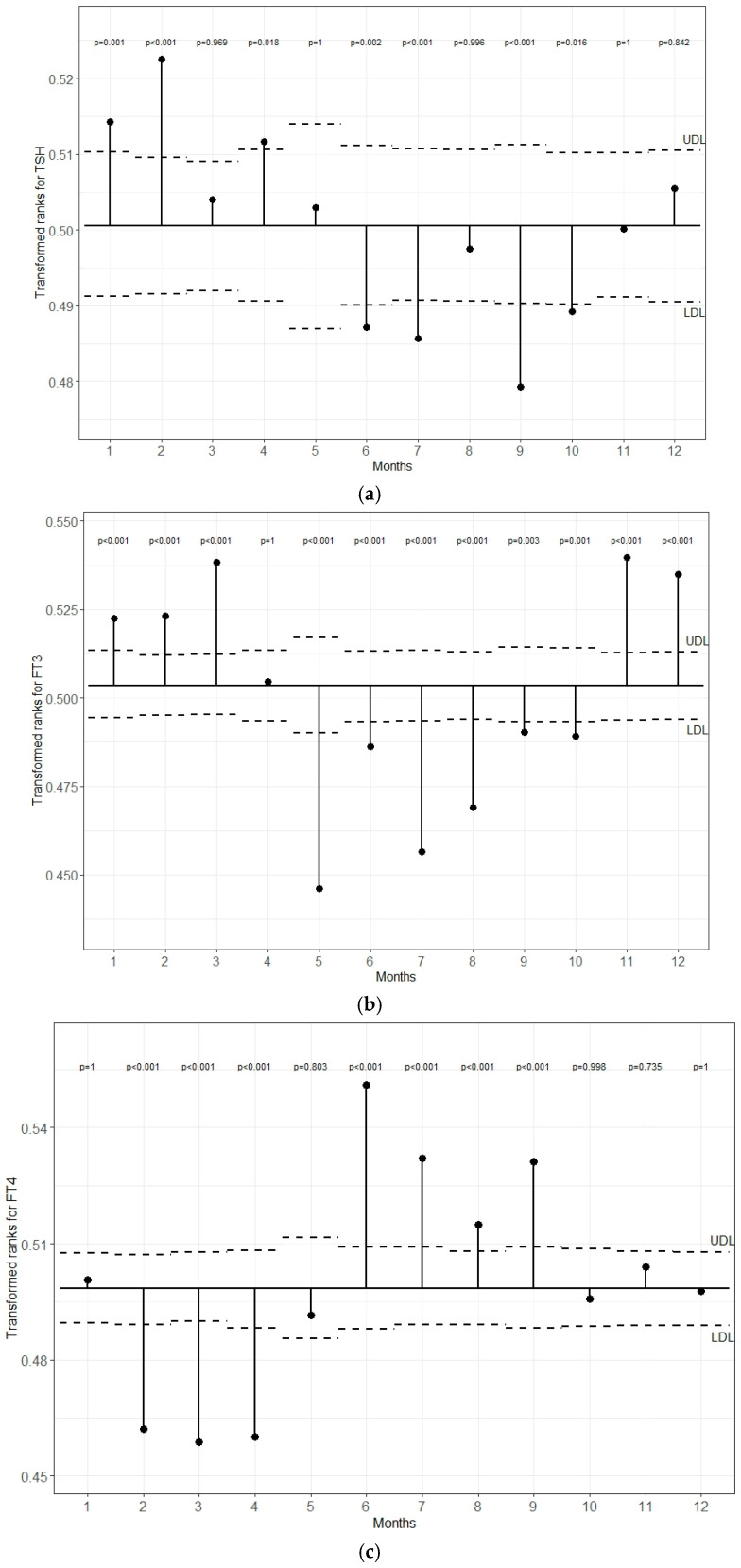
(**a**–**c**). Analysis of means (ANOM) with transformed ranks of monthly changes in the TSH, FT3, and FT4 levels, respectively.

Figure 2a–c demonstrates the transformed rank mean in each month compared with the overall mean. The plot shows that the TSH levels were significantly lower in June, July, September, and October and higher in January, February, and April than the overall transformed mean. The FT3 values from November to March were significantly higher than the overall transformed mean, and from May to October, they were significantly lower. When the FT4 values were compared each month, the levels from February to April were lower, and those from June to October were higher than the overall mean.

### 3.3. Seasonal Variations in the Median TSH, FT3, and FT4 Levels

**Figure 3:**
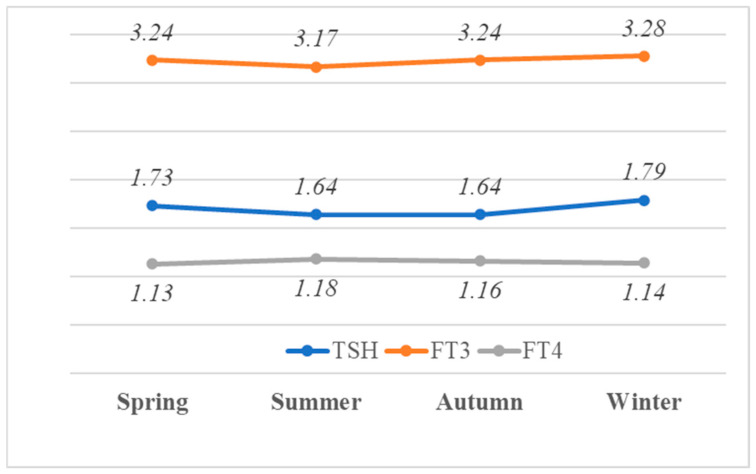
The seasonal median TSH (mIU/L), FT3 (ng/L), and FT4 (ng/dL) concentrations in each season.

The distributions of TSH, FT3, and FT4 were significantly different in at least one season from another season (all *p* < 0.001) (Figure 3). TSH and FT3 were highest in winter, while FT4 was highest in summer. Similarly, after conducting the ANCOVA, with the effect of age controlled for, it was determined that there was a significant difference in the distribution across the seasons (*p*-value < 0.001 for TSH, FT3, and FT4).

### 3.4. Transformed Rank Mean Comparison for TSH, FT3, and FT4 Levels in Each Season

**Figure 4:**
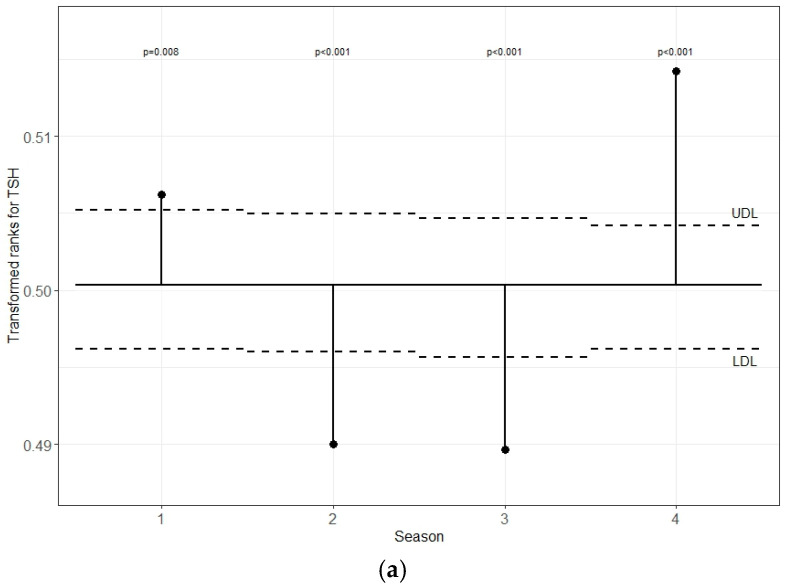

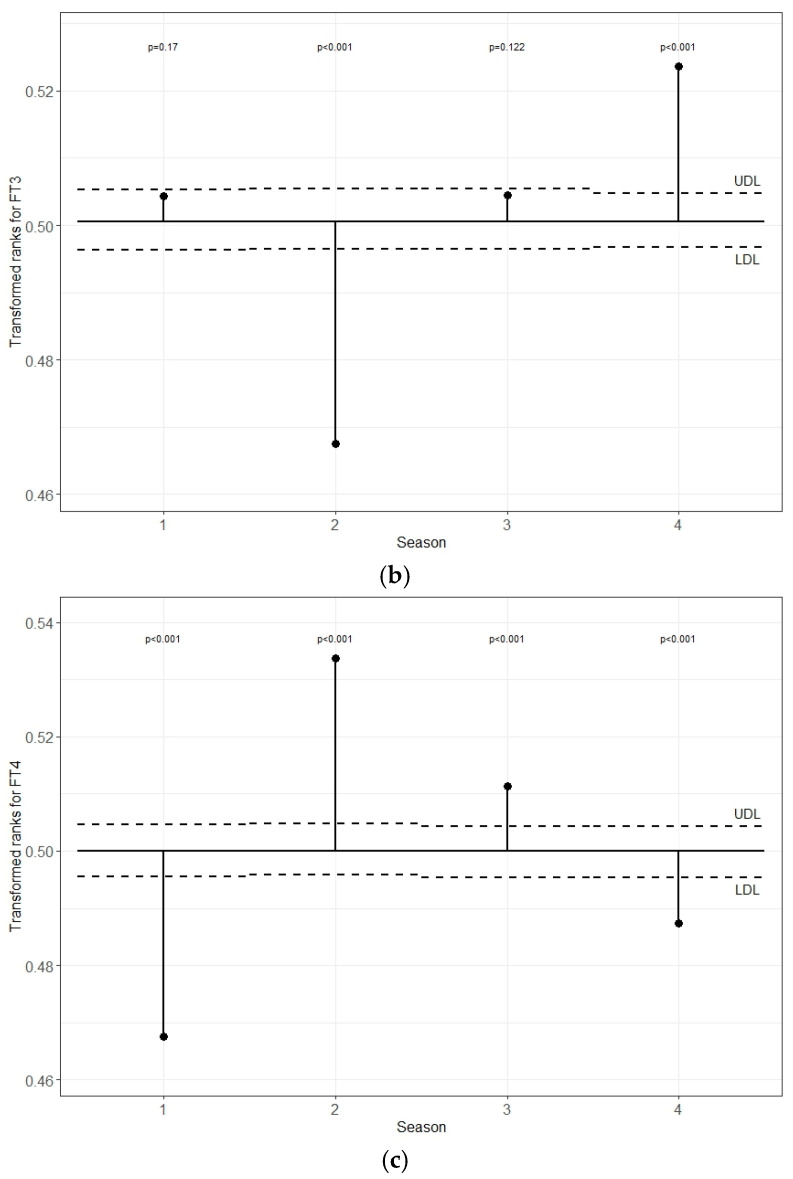
(**a**–**c**). Analysis of means (ANOM) with transformed ranks of seasonal changes in the TSH, FT3, and FT4 levels, respectively.

Figure 4a–c demonstrates the transformed rank mean in each season compared with the overall mean. The plot shows that the TSH levels were significantly lower in summer and autumn and higher in winter and spring than the overall transformed mean. The FT3 value in summer was significantly lower than the overall transformed mean and significantly higher in winter. When the FT4 values were compared in each season, the levels in summer and autumn were higher, and in spring and winter, they were lower than the overall mean.

The percentage of individuals with TSH levels below 2.5 mIU/L was higher in summer and autumn compared to the percentage in spring and winter (*p* < 0.001) (Table 1).

### 3.5. Thyroid Functional Status in Seasons

Based on the values of TSH, FT3, and FT4 levels, hyperthyroidism was detected in 22.6%, euthyroidism in 67.5%, subclinical hypothyroidism in 6.2%, and overt hypothyroidism in 3.7% of subjects. The distribution of thyroid functional status differed significantly by season (χ^2^: 58.147; *p* < 0.001). The percentages of overt hypothyroidism in the seasons were similar (*p* > 0.05). The subclinical hypothyroidism percentage was higher in winter compared to the other seasons (*p* < 0.05). The percentage of euthyroidism in spring was significantly higher than in autumn (*p* < 0.05). The rates of hyperthyroidism observed in summer and autumn were higher than those observed in spring and winter (*p* < 0.05).

## 4. Discussion

Considering the frequency and severity of thyroid dysfunctions, diagnosis, treatment, and monitoring of disease progression are very important for public health [13]. Thyroid hormones regulate various physiological processes, and their serum concentrations must be sufficient for the proper function of the whole organism, especially the reproductive system. Maternal thyroid dysfunctions may cause subfertility or infertility, menstrual irregularities, anovulation, abortion, premature delivery, and intrauterine developmental delay [2]. Therefore, TSH values in women during the reproductive period should be evaluated carefully and appropriately. One of the best indicators that can be used for this purpose is serum TSH, and knowing the factors that may affect its concentration is very important in managing patients. TSH levels are known to be affected by many factors, including age, gender, ethnicity, iodine status, and season.

Employing a big data methodology, our study explored the potential association between seasonal variations and levels of TSH and thyroid hormones in women of reproductive age. The findings revealed distinct seasonal fluctuations in TSH and thyroid hormone concentrations, with a notably higher prevalence of subclinical hypothyroidism and TSH levels exceeding 2.5 mU/l during the winter months. Notably, various clinical guidelines advocate for the treatment of pregnant women exhibiting TSH levels above 2.5 mU/l, irrespective of their antibody status, underscoring the clinical significance of these seasonal trends [14,15]. Therefore, our data should be evaluated carefully in women of childbearing age and women planning pregnancy.

To date, the sole investigation employing a big data approach to examine the relationship between seasonal variations and TSH and thyroid hormone levels in women of reproductive age was conducted by Fu et al. in China. Their study excluded individuals with thyroid dysfunction and analyzed a cohort of 48,990 women aged 20–49 between 2012 and 2018. The results demonstrated a statistically significant seasonal disparity, with the prevalence of subclinical hypothyroidism being lower in summer compared to winter (5.6% vs. 7.0%, *p* < 0.05). This finding highlights the potential influence of seasonal factors on thyroid function in this demographic [16]. While TSH, FT3, and FT4 levels were at their peak in winter, they were lowest in summer. TSH was positively correlated, whereas FT3 and FT4 were negatively correlated with age.

Studies investigating the relationship between thyroid function and seasonality have yielded conflicting results. In a large-scale study (*n* = 206,486) encompassing outpatients, inpatients, and individuals undergoing routine health check-ups in Beijing, Wang et al. observed that mean TSH levels were 1.88 μIU/L in spring, 1.86 μIU/L in summer, 1.87 μIU/L in autumn, and 1.96 μIU/L in winter. Notably, TSH levels exhibited greater variability during winter (1.96 ± 0.128 mU/L) compared to summer (1.86 ± 0.111 mU/L), suggesting a potential seasonal influence on thyroid hormone regulation. These findings underscore the complexity of the interplay between thyroid function and environmental factors [13]. Santi et al. investigated the seasonal secretion pattern of the hypothalamic–pituitary–thyroid axis between 2010 and 2017. They reported that while FT4 and FT3 did not show seasonal changes, TSH levels were higher in summer and winter (semi-annual) independently of age, sex, and environmental temperature [17].

A comprehensive meta-analysis was conducted using Review Manager 5.3 (Cochrane Library), encompassing 13 panel studies and 7 cross-sectional studies. The analysis revealed that circulating TSH levels were significantly higher during winter compared to other seasons, while FT4 levels were elevated in autumn relative to winter. In contrast, T4 levels exhibited no marked seasonal variation. Circulating T3 levels were notably higher in winter than in summer, whereas FT3 levels were lower in summer compared to autumn and spring. Further analysis of TSH seasonal dynamics (winter vs. summer) revealed gender-specific patterns: women demonstrated a pronounced increase in TSH levels during winter, whereas men did not exhibit this trend. Seasonal variations in FT3 and T4 levels were found to be independent of gender. Additionally, the seasonal dynamics of TSH remained consistent across different age groups, indicating no age-related influence on these fluctuations. These findings highlight the complex interplay between seasonality, thyroid hormone levels, and demographic factors such as gender, while underscoring the stability of certain seasonal patterns across age groups [18].

One of the possible mechanisms explaining our results could be the photoperiod. It plays a key role in regulating seasonal thyroid function. Animals in temperate climates time reproduction to optimize offspring survival. Short-incubation species like birds and small mammals breed in spring and summer as long-day (LD) breeders, while species with longer gestation, like sheep and goats, breed in autumn as short-day (SD) breeders. These animals use day length as a natural calendar to ensure offspring are born when food is plentiful and conditions are ideal. Early studies found that thyroid removal disrupted seasonal reproductive responses in birds like starlings, and restoring thyroxine reversed these effects in Japanese quail [19].

Menstrual phase can affect thyroid hormones; thyrotropes contain estrogen receptors, although they are less prevalent than in other anterior pituitary cells, and estradiol levels affect TSH responsiveness. The observed increase in serum TSH levels during pregnancy, higher TSH in women with polycystic ovary syndrome or those using estroprogestins, and elevated TSH in women of reproductive age compared to men suggest that estrogen fluctuations can influence TSH production [20]. The secretory patterns of the hormones of the hypothalamic–pituitary–gonadal axis exhibit ultradian, infradian, and circadian rhythmicity. A circannual pattern of pituitary and gonadal hormone secretion, which is established during prepuberty, becomes more pronounced in adulthood. It shows a peak in LH secretion during January (winter in the Northern Hemisphere) for both genders, and a peak in testosterone levels during late summer to autumn in men [21,22,23]. Bellastella et al. examined whether a circannual TSH rhythm exists in healthy adult men and in those with Klinefelter’s syndrome. Over three years, monthly TSH measurements revealed a significant circannual rhythm peaking in December in healthy men, while Klinefelter’s patients showed lower TSH levels and no significant rhythm [24].

The human adenohypophysis consists of two main parts: pars distalis (PD) and pars tuberalis (PT). In PT, thyrotropes lack TRH receptors, making TSH secretion independent of TRH. Instead, melatonin receptors regulate TSH release, which is triggered by photoperiodic changes. This shows that PT-derived TSH functions independently of the hypothalamic–pituitary–thyroid axis. During long days, TSH production in PT increases, stimulating deiodinase type 2 and inhibiting deiodinase type 3 in the hypothalamus. This alters thyroid hormone levels in the hypothalamus, helping control seasonal reproduction in birds and mammals.

Seasonal variations in TSH are essential, particularly in asymptomatic individuals with TSH values at the upper limit of the reference range, in that the diagnosis can change between euthyroidism and subclinical hypothyroidism. However, temporary TSH fluctuation does not necessarily mean that the patient’s thyroid function is impaired, because TSH, which might increase slightly during the cold period, may return to normal in the following hot season.

Subclinical hypothyroidism is seen in 4–18% of adults. However, 75% of patients with mild TSH elevation (TSH < 10.0 mIU/L) can become euthyroid without any treatment [25,26,27]. In a study including 422,242 subjects, it was reported that 95% of them had normal baseline TSH (0.35–5.5 mIU/L), 1.2% had decreased (<0.35 mIU/L), 3.0% had increased (>5.5 to ≤10 mIU/L), and 0.7% had excessively increased (>10 mIU/L) TSH levels. During follow-up, it was observed that the TSH levels of 346,549 patients who were not given thyroid-specific medication were normalized in 27.2%, 62.1%, and 51.2% of subjects with highly increased, increased, and decreased TSH levels, respectively [12]. Because subclinical hypothyroidism is often reversible, and similar rates of euthyroid individuals transform into subclinical hypothyroidism, multiple clinical factors should be considered before clinical decision-making. The possibility of normalization is higher, especially in young people with minimal TSH increases. These should be taken into consideration when deciding on treatment, especially in the summer–winter period, and follow-up tests should be performed [25].

Kim et al. studied seasonal effects on TSH levels and the transition between subclinical hypothyroidism and euthyroidism in 1751 subclinical hypothyroidism patients and 28,096 euthyroid individuals. Over a median follow-up of 36 months, 57.9% of subclinical hypothyroidism patients returned to euthyroidism, while 4.3% of euthyroid individuals developed subclinical hypothyroidism. TSH levels followed a biphasic pattern, increasing in winter–spring and decreasing in summer–autumn. The maximum TSH difference was 0.69 mIU/L for subclinical hypothyroidism patients and 0.30 mIU/L for euthyroid individuals. Normalization of TSH was 1.4 times more likely in summer–autumn for subclinical hypothyroidism patients, while the shift to subclinical hypothyroidism occurred 1.4 times more in winter–spring [25].

Our study has several limitations, including its retrospective design, the inclusion of data from all patients regardless of thyroid disease or medication use, and data collection from only a single hospital. Another important point is that a significant portion of the study period coincided with the COVID-19 pandemic. Due to measures such as lockdowns implemented during the pandemic, many patients remained at home, were exposed to artificial light late into the night, and experienced changes in their eating habits and sleep patterns, all of which may have influenced the measurements. Environmental factors, including photoperiod, temperature, and the use of artificial heating or cooling systems, were not controlled for in this study. Blood samples were collected indoors under constant room temperature, but it is difficult to assess real human exposure to outdoor climatic conditions. While animal studies suggest that climatic factors are significant, modern technologies (like heating and air conditioning) may reduce their impact on humans [28]. Additionally, macro TSH was not measured at this center, so the prevalence of macro TSH in the study population remains unknown. Our study has outstanding strengths. Firstly, this study was carried out in Ankara, a city that experiences all four seasons, with significant temperature variations between summer and winter. Secondly, the data collected span a continuous period of four years and include a total of 75,000 samples. Additionally, we employed a big data approach, which helps reduce the influence of confounding variables such as sex, age, individual biological differences, and thyroid dysfunctions [7].

## 5. Conclusions

This study examined the exposure–response relationship between ambient temperature and thyroid function in Turkish females of reproductive age with normal thyroid function, estimating individual temperature exposures. TSH levels increased in cold temperatures and decreased in hot temperatures, showing a negative association with temperature. These findings suggest that TSH is sensitive to temperature variations. TSH and thyroid hormones can fluctuate seasonally in women of reproductive age, with TSH levels often rising in winter. Clinicians must be cautious when interpreting thyroid function results to avoid over- or misdiagnosing thyroid disorders. Multicenter studies across various climates are needed to establish seasonally appropriate TSH values and to gain a clearer understanding of how environmental factors impact thyroid function. Given recent climate changes and health concerns, it is essential to understand the physiological responses of TSH and thyroid hormones to extreme temperatures. Further research is needed, especially for vulnerable populations like the elderly, children, and those with cardiovascular diseases.

## Figures and Tables

**Table 1:** The distribution of TSH groups in each season.

TSH Group	Season (Column %)			
	Spring	Summer	Autumn	Winter
≤2.5 mIU/L	66.1 ^a^	69.0 ^b^	68.7 ^b^	65.0 ^a^
>2.5 mIU/L	33.9	31.0	31.3	35.0

χ^2^ 100.856; *p* < 0.001. ^a,b^: Each subscript letter denotes a subset of season categories whose column proportions do not differ significantly from each other at the 0.05 level.

## Data Availability

The full dataset used in this analysis is available from the corresponding author upon reasonable request.
